# Using Musical Feedback Increases Stride Frequency in Recreational Runners

**DOI:** 10.3390/ijerph19073870

**Published:** 2022-03-24

**Authors:** Sergio Sellés-Pérez, Lara Eza-Casajús, José Fernández-Sáez, Miguel Martínez-Moreno, Roberto Cejuela

**Affiliations:** 1 Department of General and Specific Didactics, Physical Education Area, Faculty of Education, University of Alicante, 03690 Alicante, Spain; sergio.selles@ua.es (S.S.-P.); mmm146@alu.ua.es (M.M.-M.); roberto.cejuela@ua.es (R.C.); 2 Unitat de Suport a la Recerca Terres de l’Ebre, Fundació Institut, Universitari per a la Recerca a l’Atenció Primària de Salut Jordi Gol i Gurina (IDIAPJGol), 43500 Tortosa, Spain; jfernandez@idiapjgol.info; 3 Unitat de Recerca, Gerència Territorial Terres de l’Ebre, Institut Catalá de la Salut, 43500 Tortosa, Spain; 4 Facultat de Enfermería, Campus Terres de l’Ebre, Universitat Rovira i Virgili, 43500 Tortosa, Spain

**Keywords:** music feedback, stride rate, running skills, cadence

## Abstract

The number of participants in popular races has increased in recent years, with most of them being amateurs. In addition, it has been observed that there is a high percentage of injuries among them, and some of these injuries may be related to a low stride frequency. The aim of this research was to check if a continuous running training program with a musical base improves the stride frequency of popular runners. For this purpose, the effect of a 6 week continuous running training program with the help of a musical track with a constant rhythm that was 10% higher than the preferred stride frequency of the subjects was analyzed and compared to a control group that performed the continuous running training without sound stimuli. Significant increases were found in the evolution of stride frequency in the experimental group between the pre- and post-test (*p* = 0.002). No significant changes were observed in the stride frequency of the control group. These results show that training with music feedback helps to improve stride frequency in recreational runners. Future research should study the evolution of the improvement obtained in time as it is unknown if the increase in stride rate has been integrated in the runner’s technique, making the improvement obtained permanent. Future research is needed to confirm these results by enlarging the sample and carrying out an exhaustive biomechanical study.

## 1. Introduction

The number of participants in popular races has increased in the last decade, with most of these participants being amateur [1]. The 46.3% of recreational middle- and long-distance runners have suffered from injuries during one year of follow-up [2]. 

Sports injuries can be caused by multiple factors [3,4,5,6]. However, a correct support during running is essential to absorb the contact with the ground, thus avoiding the risk of injuries and preventing them [7]. Moreover, this risk increases if there is an angulation of the ankle and knee joints in the stance phase [1] caused by excessive or insufficient pronation or supination that alters the kinetic chain [7]. The most repeated injuries in runners are knee (20.9%), calf (16.3%), Achilles’ tendon (12.2%), feet (9.2%), and hip (8.8%) [2].

Stride frequency is commonly used to refer to the total number of running steps per minute [8], and the optimal stride frequency (OSF) is the one that coincides with the frequency that minimizes metabolic cost. It generally coincides with the one that runners choose that is considered as the preferred stride frequency (PSF) [9]. When runners are fatigued, the stride frequency is no longer similar to their OSF and an increase in metabolic cost takes place [10]. Running at a fixed speed can alter different aspects such as electromyography and kinetics [11].

Much research [8,12,13,14] has shown that an increase in stride frequency (SF) can reduce the risk of injury for different reasons: on the one hand, knee flexion and plantar flexion increases in the stance phase as a consequence of resistive forces are reduced, the angular velocity of these joints increases, and hip stabilization during running is improved [8]. To favor the runner’s performance, the increase has to be 10% more in SF [12,15,16].

It has been shown [13] that at the same speed, at a faster SF, the metatarsal is supported, while, during a slower SF, the forefoot and rearfoot are supported, increasing the risk of injury [8]. On the other hand, the knee, ankle, and hip joints absorb less mechanical energy [12] as the impact of the lower limbs against the ground is less [14]. In turn, there is less peak hip flexion and adduction when the loading response occurs. Changes are also observed at the ankle joint with a more plantar flexed position, and there is more knee flexion and less peak knee flexion [15,16,17]. Being able to achieve and maintain a higher-than-preferred stride frequency makes it possible to reduce lower limb loading variables, as well as the risk of reducing tibial stress fracture [14].

SF can be modified by sound stimuli and using music [18]. It has been shown that music can be a useful tool that provides various benefits, including decreased perception of fatigue [19,20], improved emotional regulation [21,22,23], the generation of adaptations at the cardiovascular and hormonal level [20,21], and improved aerobic and anaerobic exercise performance [24]. This is because musical rhythm produces a synchronous effect in physical activities with cyclical movements (running, cycling, cross-country skiing, rowing, and others) due to the synchronization of bodily movement to the beats [25,26].

In a study conducted by Atkison, Wilson, and Eubank [27], participants completed a 10 km cycling time trial. The control group listened to no music, while the experimental group listened to upbeat music. The results showed that the experimental group took significantly less time to complete the test. In addition, speed, power, and heart rate averages were also significantly higher compared to the control group.

By regulating the movement, the technical execution is perfected and the movement is more efficient [28,29,30]. This movement regulation generates a more efficient running cadence [28,31]. The motor synchronization that takes place between body movements and the rhythm of the music helps to achieve running economy as it helps runners to maintain a consistent pace [32].

A study carried out by Simpson and Karageorghis [29] showed that those who ran a 400 meters sprint race with music stimulation achieved significantly higher performance. In addition, in sports such as triathlons, the results are similar and show that those who run with synchronous music can increase exercise time [33].

In a study involving recreational runners, it was found that those who had received auditory feedback during the running test ran further than those who had not received any feedback. However, those who received both auditory and visual feedback ran further than those who had no or only visual feedback [34].

In addition, the runner can change their SF spontaneously and unconsciously after listening to sound stimuli at constant paces [35]. The increase in preferred stride frequency (PSF) should not be more than 10%, to avoid impairing running economy [12,15,16] and should not reduce running efficiency in runners [36]. It also helps to achieve better leg stiffness [37]. Such an increase must be higher than 5% because lower frequency increases have less impact on the mechanical energy absorbed by the joints [12,14,15].

Although it has been shown that an improvement in stride frequency of 5% to 10% can be produced by the perception of sound stimuli in controlled situations with little uncertainty [36], these results are different from the reality of a recreational runner. The aim of this study is to test whether running training for popular runners with music feedback at a controlled pace (+10% PSF) can lead to improvements in SF in a stable manner, increasing their PSF even in the absence of sound stimuli.

## 2. Materials and Methods

This study employed a prospective, randomized, controlled, experimental study design that was conducted for 6 weeks. All participants received written informed consent in accordance with institutional policies prior to enrolment, as well as an initial questionnaire consisting of the “PARQ & YOU” health questionnaire [38] and the “Cardiovascular Risk Factors” questionnaire [39]. Methods used in this research were revised and approved by the Ethics committee of Alicante University (UA-2017-04-11-Expedient).

In addition, questionnaires on sports history, injury history, and musical tastes were included, which were intended to collect information about the participants, but in no case served as study variables.

The nature of the study aimed to be as noninvasive as possible in the runners’ training routines. Therefore, the methodology of the study tried to adapt to the real situations of the runners, respecting the natural training places of each athlete, as long as they were on a 0 ± 1.0% slope.

### 2.1. Sample and Procedure

Twenty uninjured runners (11 men, 9 women) were initially recruited through local clubs and friends. To be part of the study group, the following inclusion criteria were considered: to be of legal age, to be an active runner, and to run more than 15 km per week. The exclusion criteria were the following: suffering or having suffered musculoskeletal injuries in the previous 6 months, suffering from any illness that causes pain when running, pregnant women, inability to run, having any peripheral cardiovascular disease, having any cardiovascular or neurological disease, having 2 or more risk factors as established by the American College of Sports Medicine [39], and failure to pass the 30 s tempo run test.

Participants attended a meeting to be informed, to complete the questionnaires and the informed consent, and to measure their weight, BMI, and height. Measurements were performed following the protocol of the International Society for the Advancement of Kineatropometry (ISAK) [40]. A Seca^®^ measuring rod model 213 with a sensitivity of 1 mm and a Tanita^®^ digital scale model BC545N with an accuracy of 100 g were used for the measurements.

Once the initial test was performed and the PSF of all participants was analyzed, 8 participants dropped out of the research (4 of them reported injuries and 4 did not have time availability). The research finally included 12 runners (8 males, 4 females) with an average age of 36.7 years (±6.5), height of 1.73 m (±0.08), weight of 70.2 kg (±13.5), and a BMI of 23.3 (±2.9). Seven were assigned to the experimental group and five to the control group depending on the results obtained in the music test. No significant differences in age, height, weight, or BMI were observed between the two groups (Table 1):

### 2.2. Protocol of the Test to Determine Training Improvements

After the meeting and completion of all questionnaires and informed consent, participants were called for the initial test.

The subjective perception of effort measured by means of the Borg scale in its CR-10 version [41] was established as a training control. It was also emphasized to the participants the importance of maintaining the same pace in all training sessions and tests, because at different speeds, the runner’s SF can oscillate.

The initial test was the same for all subjects. It started with a dynamic warm-up of 5–10 min self-selected by the participants. Subsequently, a music rhythm test was performed to assess the participants’ ability to keep the rhythm of the music while running. The participants were asked to run on the spot following the different music rhythms. They had to make ground contact by matching each foot contact with a beat of the music, or in the case of double beats, make two ground contacts for each beat of the music. The beats of the music measured in beats per minute (bpm) were 120, 60, 60 double, 140, 70, 70 double, 160, 80, and 80 double. Each beat of the music was played for 30 s. The participant had to acquire the running rhythm spontaneously and without instructions. The rhythm was paced using a digital metronome provided by the mobile application called “Soundbrenner.”

Afterward, the test to measure PSF was performed. Participants were asked to run for 20 min at an intensity of 5/6 (moderate intensity) of subjective perception of effort, measured by the Borg Test in its CR-10 version [41]. At the end of this time, participants were instructed to perform a self-selected cool-down.

The test was performed on a 450 m straight, 0% gradient asphalt road, in an attempt to simulate the natural running conditions of amateur runners. In the middle of the course, a digital marker was placed at a point indicating 50 m. During the agreed time, the runners had to run laps around the course.

From the 10th minute of the test, each participant was recorded at least 3 times when passing the established point, with an approximately 2 min difference between recordings. The recording was made with a mobile device with a video capture configuration of 60 FPS, in a sagittal plane perpendicularly at a distance of 30 m from the course. Subsequently, the videos were analyzed with Kinovea^®^ software (Joan Charman & Contrib, Bourdeaux, France), where 10 steps (20 supports) were marked and the time elapsed in those 10 steps was measured. This was performed with all the shots, and by calculating the average of all of them, the PSF of each runner was obtained using the formula:

(1) $$PSF\;\left(\mathrm{steps}/\min\right)=\frac{60^{\prime \prime }\times 10\;\mathrm{steps}}{\mathrm{average}\;{\mathrm{time}}^{\prime \prime }}$$

Formula (1) to obtain the PSF of each runner.

This test was performed 3 times. The mid-test was performed 15 days after the pre-test, and the post-test was performed 15 days after the mid-test. The musical rhythm test was only performed in the initial test. The OSF did not change during the study, always remaining the same as that obtained in the initial test.

**Figure 1:**
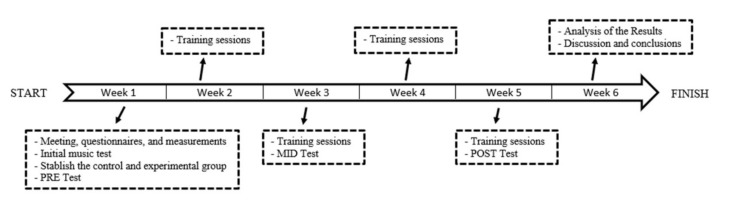
Research’s timeline.

Figure 1 shows a general the timeline of the research.

### 2.3. Creation of Music Tracks

With the data obtained in the initial test on the OSF, music tracks were elaborated using the music editing software Ableton live 9^®^ (Ableton, Berlin, Germany). The musical tastes of the participants were respected. The music tracks contained between 18 and 22 songs that were mixed without altering or interrupting the rhythm. The speed of all of them was modified by unifying the speed of the track, which was constantly equal to the OSF of each subject. 

### 2.4. Experimental and Control Group Training

The experimental group received an individualized music track that matched their OSF in speed (bpm). They were asked to follow their natural training plan of continuous running, which had to comply with the established premises: more than 15 km/week and at an intensity of 5/6 on the Borg CR-10 scale [41].

The participants were instructed to perform the training sessions by trying to follow the pace of the stride frequency that was marked by an audio device, chosen by the participants, which played the personalized music track. At the end of the training session, they were asked to record the date, distance covered, total running time, and difficulties encountered in following the musical rhythm in a record table.

Every two weeks, they attended the stride frequency test to observe the evolution of their stride frequency, where they were not allowed to carry audio devices. In this way, it was possible to observe the persistence of the effects of music-based stride frequency training. The control group followed their normal training plan as planned. In addition, they were asked to record the date, distance run in meters, and total running time on a recording chart.

As with the experimental group, participants of the control group were scheduled every two weeks to observe the evolution of their stride frequency, which was compared with the results of the experimental group.

### 2.5. Statistical Analysis

A descriptive analysis of the sample with the mean, standard deviation, and median was carried out. The nonparametric Mann–Whitney U test was used to detect statistically significant differences between the two groups. The nonparametric Wilcoxon test was used to detect statistically significant differences within each group. Then, the size of the nonparametric effect and the probability of superiority were used to quantify this difference [42,43]. The level of statistical significance established was *p* = 0.05. The statistical program SPSS 20.0 and a Microsoft Excel spreadsheet were used to record and analyze the data.

## 3. Results

Table 2 shows the characteristics of the control and experimental groups. At the beginning of the intervention, both groups had a very similar mean cadence in the PRE test. No significant differences were found between the groups (*p* = 0.935).

Looking at Table 3, it can be seen that there is a large improvement index between the experimental group and the control group in the PRE test, in the MID test, and in the total improvement data as the nonparametric effect size was high in all cases (r > 0.8).

Table 4 shows the distribution of tests by group. Considering the cadence of the PRE Test in both the control and experimental groups, the median of the control group was 82.91 spm, lower than that of the experimental group (83.03 spm). Although no significant differences were found (*p* = 0.935), the effect size was found to be small between the two groups (r = 0.023 < 0.2).

Regarding the MID Test cadence, there was still no significant difference between the groups (*p* = 0.062), although the difference between the medians was higher than that of the PRE Test (control group 82.91 spm and experimental group 84.63) with a medium effect size (r = 0.530 > 0.5).

In the POST test, significant differences could be found between the two groups (*p* = 0.004 **), the size of the nonparametric effect was high (r = 0.822 > 0.8), and the PS value was 0.000, so there is a very high probability that the test values of a randomly selected athlete in the experimental group would be higher than the values of the athletes in the control group.

Table 5 shows the comparison within each group of the results obtained. On the one hand, in the control group, no significant differences were found between the tests, as, in none of the cases, *p* < 0.05. On the other hand, in the experimental group, significant differences were found between all the tests compared, with *p* < 0.05 in all cases.

**Figure 2:**
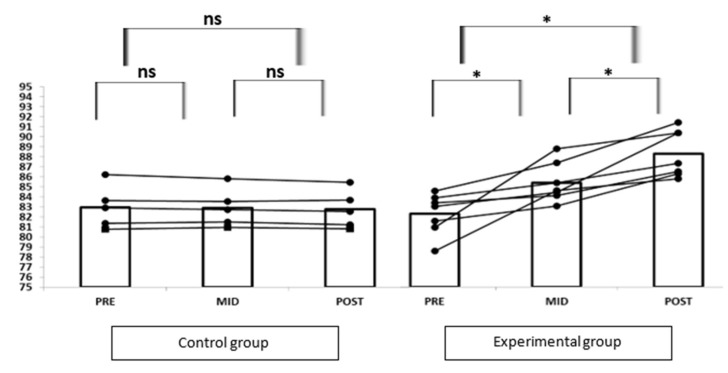
Graph of cadence evolution in the control group and the experimental group. The nonparametric Wilcoxon test was used for the detection of significant difference between the tests (* *p* < 0.05). No significant difference was found between the tests (ns: *p* > 0.05). Data are presented individually for each participant and as overall mean.

The individual response of each subject is shown in Figure 2. On the one hand, there were no significant differences between the results of the athletes in the control group in the different tests (*p* > 0.05). On the other hand, the results obtained in the experimental group showed significant differences not only between the PRE and POST test (*p* < 0.05) but also between the PRE and MID test. (*p* < 0.05). There were no appreciable changes in the subjects of the control group between tests. Meanwhile, all subjects increased their cadence between tests in the experimental group.

## 4. Discussion

The goal of the study was to test whether music feedback training with a controlled pace (+10% PSF) would lead to improvements in stride frequency and that this would be stable over time, leading to an increase in the preferred stride frequency. It has been shown that for music to be effective as an aid to improve performance, efficiency, and motivation, it must be used in low aerobic physical activities at a low and medium intensity, not in those where athletes reach the anaerobic threshold [8,11,20]. Thus, the participants of our research performed the test and their training sessions mainly at moderate intensity.

The results revealed that there was indeed an improvement in stride frequency in the experimental group, which used music feedback during their continuous running training sessions. Due to the increase in SF, the risk of injury can be reduced as the resistive forces are reduced and, among other aspects, an improvement of hip stabilization during running takes place [8,12,13,14]. In addition, the use of continuous music-based biofeedback helps to reduce tibial shock [14,44]. In addition, the use of music becomes a stimulus that favors the learning of running technique and consequently reduces the risk of injury [8,12,13,14,45].

Van Dyck and others [35] introduced music as a stride pace-modifying strategy in their study, and music was shown to have a spontaneous effect on SF modification, as runners modified their stride pace with imperceptible changes in music tempo, and without being informed of the change, runners increased their SF when the music increased their pace and decreased their SF when the music carried a slower tempo. 

Baumgartner’s study [36] demonstrated that it is possible to increase the stride frequency of subjects by 5% to 10% by carrying a sound stimulus at a constant tempo, although the sound stimulus was constant beeps emitted by a clock that was unmotivating and even stressful for the runners. Numerous studies have demonstrated the importance of music as a tool that favors the motivation of athletes and consequently their performance [28,29,46,47].

Listening to music during running has different effects on both running speed and heart rate [46,48,49]. It also improves performance [46,47], and interactions with tempo music are associated with different performance benefits [29] such as lengthening the time to exhaustion, accelerating the rate of post-exercise recovery [20,24], and increasing work time [48,49]. Depending on the style of music used, different benefits can be achieved [47]. On the one hand, listening to fast music during exercise helps to increase self-paced intensity and improve exertion capacity. On the other hand, listening to slow music is more commonly used after exercise for the purpose of accelerating the rate of recovery.

Scientific evidence shows that listening to music during sports practice is associated with many positive effects both physiologically and psychologically [28]. On the one hand, at the physiological level, it helps to decrease the perception of fatigue [19,20] and the generation of adaptations at the cardiovascular and hormonal level [20,21].

On the other hand, at the psychological level, it can serve as a tool that helps to regulate emotions, effectively having implications on the athlete’s motivation [20,21,22,23,24,35]. Consequently, it becomes a tool that helps to improve performance [20,24] and is considered a pleasant stimulus [44].

Both men and women participated in this research, and it is also necessary to consider some biomechanical differences between genders because of the anthropometric and physical characteristics of each gender. Women have a higher stride frequency because their leg length is shorter, and their stride width is smaller [50] and they have a higher peak hip adduction, hip internal rotation, and knee abduction angle compared to men. They also show significant differences in hip frontal and transverse plane negative work [51].

## 5. Conclusions

The present study had some limitations that warrant a brief discussion. First, we acknowledge that the study was performed with a reduced sample, and it would be very interesting to enlarge the sample to amplify the results obtained.

It would also be interesting to carry out an exhaustive biomechanical study to measure different forces and angles to examine the running technique used and the changes in these parameters after the use of music feedback. Finally, it would have been of great interest to have carried out an evaluation after a certain period (for example, a month) without using the feedback to check whether the effects of the intervention are maintained in the long term.

At the end of the research, it was found that there were significant improvements in the experimental group in the different tests, while the differences between tests in the control group were not significant. The results showed that training with music feedback helps to increase stride frequency in recreational runners. This fact may be of great interest to coaches as a simple tool as a music playlist with a preset bpm can improve biomechanical parameters of running technique. In addition, this tool will be motivating for recreational runners. However, future research is needed to confirm these results.

## Figures and Tables

**Table 1:** Participants’ characteristics.

	Control Group (*n* = 5) Average (SD)	Experimental Group (*n* = 7) Average (SD)	^a^ *p*
Age (years)	38 (7.30)	35 (5.91)	0.37
Height (m)	1.73 (0.09)	1.72 (0.08)	0.87
Weight (kg)	71.76 (12.34)	69.00 (15.20)	0.57
km per Week	31 (11.94)	27 (12.67)	0.74
Gender	Male *n* = 4; Female *n* = 1	Male *n* = 4; Female *n* = 3	

SD = Standard deviation. ^a^ Nonparametric Mann–Whitney U test.

**Table 2:** Baseline description of the sample according to control group or experimental group.

	Control			Experimental			
	Average	SD	Median	Average	SD	Median	^a^ *p*
Age	38.88	7.36	41.60	35.07	5.75	33.30	0.372
Size (m)	1.73	0.09	1.74	1.72	0.08	1.73	0.871
Weight (kg)	72.00	12.00	72.00	69.00	15.00	63.00	0.569
BMI	23.84	3.13	23.30	23.01	2.88	22.90	0.745
km/week	22.00	6.00	20.00	23.00	6.00	22.00	0.739
PRE rhythm test	7.24	0.18	7.24	7.29	0.19	7.26	0.685
MID rhythm test	7.24	0.17	7.23	7.29	0.20	7.21	1.000
POST rhythm test	7.23	0.21	7.24	7.31	0.20	7:30	0.745
Average PRE rhythm test	7.24	0.19	7.24	7.29	0.19	7.23	0.935
Cadence PRE rhythm test	82.97	2.15	82.91	82.29	2.07	83.03	0.935

SD = Standard deviation. ^a^ Nonparametric Mann–Whitney U test.

**Table 3:** Distribution of the improvement index of the three according to the group. Effect size and probability of superiority.

	Control			Experimental							
	Average	SD	Median	Average	SD	Median	^a^ *p*	^b^ Z	^c^ U	^d^ r	^e^ PS
Improvement Index PRE test	−0.09	0.29	−0.14	3.85	3.45	1.80	0.003 **	−2.85	4	−0.82	0.11
Improvement Index MID Test	−0.17	0.22	−0.23	3.41	1.96	2.88	0.003 **	−2.84	0	−0.82	0.00
Total Improvement	−0.26	0.39	−0.14	7.36	2.92	8.13	0.003 **	−2.84	0	−0.82	0.00

SD = Standard deviation. ^a^ Nonparametric Mann–Whitney U test. ^b^ Standardized Mann–Whitney U value. ^c^ Mann–Whitney U. ^d^ Size of the nonparametric effect. ^e^ Probability of superiority. ** *p* < 0.01.

**Table 4:** Distribution of the tests according to the group.

	Control			Experimental							
	Average	^to^ ds	Median	Average	^to^ ds	Median	^a^ *p*	^b^ Z	^c^ U	^d^ r	^e^ PS
PRE test rhythm 1	7.24	0.18	7.24	7.29	0.19	7.26	0.685	−0.40	15	−0.11	0.43
PRE test rhythm 2	7.24	0.17	7.23	7.29	0.20	7.21	1.000	0.00	17.5	0.00	0.50
PRE test rhythm 3	7.23	0.21	7.24	7.31	0.20	7:30	0.745	−0.32	15.5	−0.09	0.44
Average PRE Test	7.24	0.19	7.24	7.29	0.19	7.23	0.935	−0.08	17	−0.02	0.48
Cadence Pre Test	82.97	2.15	82.91	82.29	2.07	83.03	0.935	−0.08	17	−0.02	0.48
MID test rhythm 1	7.22	0.18	7.25	7.06	0.17	7.13	0.223	−1.22	10	−0.35	0.28
MID test rhythm 2	7.28	0.20	7.36	7.01	0.16	7.06	0.042 *	−2.03	5	−0.58	0.14
MID test rhythm 3	7.22	0.17	7.25	7.01	0.17	7.06	0.143	−1.46	8.5	−0.42	0.24
Average MID test	7.24	0.17	7.25	7.03	0.16	7.09	0.062	−1.87	6	−0.54	0.17
Cadence MID test	82.89	1.92	82.72	85.41	2.01	84.63	0.062	−1.87	6	−0.54	0.17
POST Test rhythm 1	7.25	0.16	7.27	6.79	0.26	6.98	0.006 **	−2.77	0.5	−0.80	0.01
POST Test rhythm 2	7.28	0.15	7.29	6.81	0.15	6.89	0.004 **	−2.86	0	−0.82	0.00
POST Test rhythm 3	7.23	0.20	7.27	6.80	0.19	6.87	0.007 **	−2.68	one	−0.77	0.03
Average POST test	7.25	0.16	7.27	6.80	0.18	6.87	0.004 **	−2.84	0	−0.82	0.00
Cadence POST test	82.75	1.89	82.53	88.32	2.35	87.34	0.004 **	−2.84	0	−0.82	0.00

^to^ Standard deviation. ^a^ Nonparametric Mann–Whitney U test. ^b^ Standardized Mann–Whitney U value. ^c^ Mann–Whitney U. ^d^ Size of the nonparametric effect. ^e^ Probability of superiority. * *p* < 0.05. ** *p* < 0.01.

**Table 5:** Comparison within each group of the results in the PRE Test, MID Test, and POST Test.

		Half	^to^ ds	Median	Half	^to^ ds	Median	^a^ *p*	^b^ Z	^c^ r
Control	Contrast PRE with MID	82.97	2.15	82.91	82.89	1.92	82.72	0.917	−0.10	−0.03
	Contrast MID with POST	82.89	1.92	82.72	82.75	1.89	82.53	0.841	0.84	0.26
	Contrast PRE with POST	82.97	2.15	82.91	82.75	1.89	82.53	0.917	0.92	0.29
Experimental	Contrast PRE with MID	82.29	2.07	83.03	85.41	2.01	84.63	0.011 *	0.01	0.003
	Contrast MID with POST	85.41	2.01	84.63	88.32	2.35	87.34	0.038 *	0.04	0.01
	Contrast PRE with POST	82.29	2.07	83.03	88.32	2.35	87.34	0.002 **	0.002	0.001

^to^ Standard deviation. ^a^ Wilcoxon nonparametric test for related samples. ^b^ Standardized Wilcoxon W value. ^c^ Size of the nonparametric effect. * *p* < 0.05. ** *p* < 0.01.

## Data Availability

All relevant data are within the manuscript.
